# IMPACT OF GENDER FOR ELDER MISTREATMENT WITHIN DEMENTIA FAMILY CAREGIVING

**DOI:** 10.1093/geroni/igae098.0496

**Published:** 2024-12-31

**Authors:** Carolyn Pickering, Leila Wood

**Affiliations:** Chair; Discussant

## Abstract

Gender plays a significant role in interpersonal violence throughout life, particularly through gender-based violence or violence driven by gender-based power dynamics. However, understanding the role of gender in elder mistreatment within dementia family caregiving is more complex due to the various layers affected by gender. For instance, societal gender norms often lead to women being primary caregivers, and trends in dementia mean women are more likely to be care recipients. Gender influences access to caregiver resources, such as financial support, and impacts outcomes like caregiver depression. This symposium aims to explore the diverse ways gender influences elder mistreatment in family caregiving contexts. The first paper investigates how the gender of the caregiver influences the likelihood of engaging in various subtypes of elder mistreatment. The second paper delves into how polysubstance use affects the risk of different types of elder mistreatment, and how this risk varies based on caregiver gender. The third paper examines perceived reciprocity as a protective factor for elder mistreatment subtypes and explores whether it plays a stronger role based on caregiver gender. The last paper utilizes a gerofeminist lens to examine the intersection of gender and physical disability in the risk of being victimized by various subtypes of elder mistreatment.

